# Bioreactor Suspension Culture: Differentiation and Production of Cardiomyocyte Spheroids From Human Induced Pluripotent Stem Cells

**DOI:** 10.3389/fbioe.2021.674260

**Published:** 2021-06-11

**Authors:** Asher Kahn-Krell, Danielle Pretorius, Jianfa Ou, Vladimir G. Fast, Silvio Litovsky, Joel Berry, Xiaoguang (Margaret) Liu, Jianyi Zhang

**Affiliations:** ^1^Department of Biomedical Engineering, School of Medicine and School of Engineering, University of Alabama at Birmingham, Birmingham, AL, United States; ^2^Division of Anatomic Pathology, Department of Pathology, University of Alabama at Birmingham, Birmingham, AL, United States; ^3^Department of Medicine/Cardiovascular Diseases, University of Alabama at Birmingham, Birmingham, AL, United States

**Keywords:** pluripotent stem cell, cardiomyocyte, suspension culture, maturation, robust scale-up

## Abstract

Human induced-pluripotent stem cells (hiPSCs) can be efficiently differentiated into cardiomyocytes (hiPSC-CMs) via the GiWi method, which uses small-molecule inhibitors of glycogen synthase kinase (GSK) and tankyrase to first activate and then suppress Wnt signaling. However, this method is typically conducted in 6-well culture plates with two-dimensional (2D) cell sheets, and consequently, cannot be easily scaled to produce the large numbers of hiPSC-CMs needed for clinical applications. Cell suspensions are more suitable than 2D systems for commercial biomanufacturing, and suspended hiPSCs form free-floating aggregates (i.e., spheroids) that can also be differentiated into hiPSC-CMs. Here, we introduce a protocol for differentiating suspensions of hiPSC spheroids into cardiomyocytes that is based on the GiWi method. After optimization based on cardiac troponin T staining, the purity of hiPSC-CMs differentiated via our novel protocol exceeded 98% with yields of about 1.5 million hiPSC-CMs/mL and less between-batch purity variability than hiPSC-CMs produced in 2D cultures; furthermore, the culture volume could be increased ∼10-fold to 30 mL with no need for re-optimization, which suggests that this method can serve as a framework for large-scale hiPSC-CM production.

## Introduction

The experimental manipulation of stem/progenitor cells has led to continuous improvements in cell viability, differentiation efficiency, and functional activity (Laco et al., 2018; Le et al., 2018; Biermann et al., 2019; Cai et al., 2019; Leitolis et al., 2019; Valls-Margarit et al., 2019; Yang et al., 2019; Zhu et al., 2020). Contemporary protocols for differentiating human induced-pluripotent stem cells (hiPSCs) into cardiomyocytes (hiPSC-CMs) are often based on the GiWi method, which uses small-molecule inhibitors of glycogen synthase kinase (GSK) and tankyrase to alternately activate and then suppress the Wnt signaling pathway (Mazzola and Pasquale, 2020). The remarkable efficiency of the GiWi method has relieved the scarcity of cardiomyocytes for research applications and, consequently, has profoundly impacted the development of cell-based cardiac therapies, including implantable engineered cardiac-tissue patches and hiPSC-CM–derived cell products (e.g., exosomes), as well as in-vitro models for mechanistic studies and drug development (Jackman et al., 2018; Liu et al., 2018; Meyer et al., 2019; Mills et al., 2019; Noor et al., 2019; Yeung et al., 2019; Gao et al., 2020; Pretorius et al., 2020). However, because the GiWi method is typically used to differentiate two-dimensional (2D) cell sheets in 6-well culture plates (Sharma et al., 2015), it may not be sufficiently scalable to produce the number of hiPSC-CMs needed for high-throughput cardiotoxicity assessments or for clinical applications such as the treatment of acute myocardial infarction (MI), which often results in the loss of ∼1 billion cardiomyocytes (Chong et al., 2014; Kropp et al., 2016; Dunn and Palecek, 2018). Higher yields may be achievable with multilayered/stacked flasks or multicarrier-based systems, but neither of these methods have been fully scaled, and both require materials and reagents that are not readily available (Ting et al., 2014, 2018; Breckwoldt et al., 2017; Le and Hasegawa, 2019; Chang et al., 2020; Laco et al., 2020).

When cultured in a three-dimensional (3D) environment, hiPSCs form free-floating suspensions of aggregated cells (i.e., spheroids) that can also be differentiated into hiPSC-CMs with tools that are both widely available and well-characterized. Several groups have used this approach to generate up to 1-L volumes of cardiomyocytes that are more than 90% pure (Chen et al., 2015; Fonoudi et al., 2015; Kempf et al., 2015; Halloin et al., 2019; Hamad et al., 2019), and some evidence suggests that suspension-differentiated hiPSC-CMs may be more mature and, consequently, more suitable for clinical applications, than hiPSC-CMs generated via 2D differentiation protocols (Jeziorowska et al., 2017; Correia et al., 2018). Here, we introduce a novel protocol for differentiating suspensions of hiPSC spheroids into cardiomyocytes that serves as a framework for further scale up to produce the large number of hiPSC-CMs required for clinical applications. We chose a shaker flask based system for this study for both its simplicity and wide availability. This will allow broad application for researchers without advanced bioreactor experience and equipment to expand their culture volumes. However, further scale up for biomanufacturing purposes will require additional optimization and engineering to account for multiple factors including but not limited to oxygen and gas diffusion, mixing and shear stress, as well as temperature and pH monitoring (Amit et al., 2011; Shafa et al., 2011; Abbasalizadeh et al., 2012; Lattermann and Büchs, 2016; Zweigerdt et al., 2016). Our protocol is based on the GiWi method and was optimized for maximum purity and yield by manipulating the initial cell density, reagent concentrations, and other culture conditions. The differentiated hiPSC-CMs were also thoroughly characterized via morphological assessments and by monitoring the expression of cardiomyocyte-specific genes (including maturity markers).

## Materials and Methods

### hiPSC Culture and Differentiation

The University of Minnesota Human Subjects Research Institutional Review Board approved all protocols related to cell line establishment in this study. The hiPSCs used in this study were generated from cardiac fibroblasts as previously reported (Zhang et al., 2018) and maintained on Geltrex-coated (Gibco) 6-well plates in mTesR Plus medium (STEMCell Technologies, Canada) with daily medium changes until 90–100% confluent and then prepared for the differentiation protocol over 7 days (i.e., beginning on day-7). The cells were washed once with Dulbecco’s Phosphate-Buffered Saline (DPBS) and incubated with 0.5 mL Gentle Cell Dissociation Reagent (GCDR; STEMCell Technologies) for 6 min at 37°C; then, the GCDR was aspirated, and 1 mL TeSR E8 3D medium (STEMCell Technologies) supplemented with 10 μM Y-27632 (BD Biosciences Cat# 562822, RRID:AB_2869435) was gently pipetted into each well to dislodge the cells and disaggregate them into small clumps. Cells from all 6 wells per plate were collected, suspended in 40 mL of TeSR E8 3D Seed medium (STEMCell Technologies), placed in a 125-mL shaker flask (Fisher Scientific), and cultured on a Belly Dancer Shaker (IBI Scientific) at 70 rpm with 5% CO_2_ at 37°C. On days-6 and 5, 1.2 mL TeSR E8 3D Feed medium (STEMCell Technologies) was added to the culture flasks, and the cells were passaged on day-4. Passaging was performed by disaggregating the cells into smaller clumps and transferring them into a final volume of 80 mL TeSR E8 3D Seed medium; then, the cells were cultured at 50 rpm with daily additions of 2.4 mL TeSR E8 3D Feed medium until day-1, when half of the culture medium was replaced with fresh TeSR E8 3D Seed medium.

Differentiation was initiated on Day 0, and the protocol was optimized by varying the initial cell density (0.26, 0.67, 1.1, 1.6, 2.1, and 5.1 10^6^ cell/mL), CHIR99021 concentration (4, 5, 6, 7, 8, and 9 μM), and shaking speed (0, 20, 55, and 75 rpm). Briefly, 1 mL of the hiPSC-spheroid suspension was collected; then, the cells were dissociated with GCDR and counted to calculate the cell density. The remaining spheroids were washed through a 500 μm filter and collected on a reversible 40 μm filter (pluriSelect) to establish a homogenous population prior to differentiation. The spheroids were washed out with RPMI 1640 supplemented with 1 B27 without insulin (RPMI/B27–) and CHIR99021 with a final volume of 2.5 mL in low attachment 6 well plates (Corning) for optimization and 30 mL in 125 mL flasks (Thermo Fisher Scientific) for subsequent experiments. Twenty-four hours later (i.e., on day 1) the medium was replaced with fresh RPMI/B27–, and the culture volume was increased by 20% and maintained at 1.2 the initial volume for all subsequent medium changes. On day 3, half the medium was replaced with RPMI/B27– containing 10 μM IWR-1, and on day 5, the medium was completely refreshed with RPMI/B27–. On day 7, the medium was completely changed to RPMI 1640 supplemented with B27 with insulin (RPMI/B27+), and the cells were cultured for two more days until day 9, when the differentiated hiPSC-CMs cells were purified. Purification was performed via metabolic selection: the medium was completely changed to glucose-free RPMI 1640 supplemented with B27 with insulin and 0.12% sodium DL-lactate, and the cells were cultured for 72 h until day 12, when the media was changed back to RPMI/B27+. The purified hiPSC-CMs were maintained in RPMI/B27+ with partial medium changes every 3 days.

### Flow Cytometry

Cells were dissociated into single cells via treatment with cardiomyocyte dissociation media (CMDM, STEMCell Technologies) for 10–20 min at 37°C, resuspended in cardiomyocyte support media (STEMCell Technologies), counted, centrifuged at 300 g for 3 min, washed with DPBS, fixed in 4% paraformaldehyde (PFA) for 20 min, permeabilized with 0.1% Triton-X, blocked with 4% bovine serum albumin (BSA) in 4% fetal bovine serum (FBS), stained with Zenon conjugated anti-Troponin T antibody or primary antibody (Supplementary Table 1), incubated for 60 min, and washed with DPBS. Around 5 × 10^5^ cells were used per sample with spheroids from individual batches analyzed as a single sample. Analysis on an Attune NxT Flow Cytometer (Thermo Fisher) used lasers FSC, SSC, and BL1 with voltages of 80, 310, and 260, respectively. FlowJo (FlowJo, RRID:SCR_008520) was used to gate the single cell population and a threshold was set at 1.3 × 10^3^ volts for cardiac troponin T positivity with consistent gating used across samples. Each marker was examined in at least 4 independent batches of cells.

### Real-Time Quantitative Reverse-Transcription Polymerase Chain Reaction (RT-qPCR)

Cells were lysed with TRIZOL (Thermo Fisher), and lysates were homogenized by repeatedly drawing/expelling them into/from a pipette; then, the RNA was purified in Direct-zol^TM^ RNA MiniPrep Plus columns (Zymo Research) and treated with DNAase I. Reverse transcription was performed with SuperScript^TM^ IV VILO^TM^ Master Mix (Thermo Fisher) as directed by the manufacturer’s protocol, and samples (5 ng with 500 nM primers; Supplementary Table 2) were analyzed on a QuantStudio 3 Real-Time PCR System (Applied Biosystems) with PowerUp SYBR Green Master Mix (Applied Biosystems). Measurements were quantified via normalization to measurements of glyceraldehyde phosphate dehydrogenase (GAPDH) RNA abundance in the same sample. Each marker was examined in at least 4 independent batches of cells with GAPDH replicates used to account for measurement error.

### Western Blotting

Protein lysates were collected by treating cells with RIPA buffer (Thermo Fisher Scientific) supplemented with HALT Proteinase Inhibitor (Thermo Fisher Scientific) and homogenized via pipetting. Total protein concentrations were calculated via BCA assay (Fisher Scientific); then, 6 μg of each sample was loaded onto a 4–20% Mini-PROTEAN^®^ TGX^TM^ Precast Protein Gel (Biorad) and run at 100 V for 1 h. Samples were transferred to a nitrocellulose membrane by using the Trans-Blot Turbo System (Biorad), blocked in 5% milk, and then incubated with primary antibody (Supplementary Table 1) overnight at 4°C and with secondary antibody for 1 h at room temperature. ECL Chemiluminescent Reagent (GE Healthcare Amersham) was applied to the membrane for 5 min, and then the membrane was exposed on the ChemiDoc Touch Imaging System (Biorad). Each marker was examined in at least 4 independent batches of cells.

### Tissue Preservation

Samples were fixed in 4% formaldehyde (Pierce, Thermo Fisher Scientific, # 28906) for 1 h and then embedded in either optimal cutting temperature (OCT) compound (Fisher Health Care, United States) or paraffin for histological analysis.

### Histochemistry

Sections (10-μm) were deparaffinized, rehydrated, stained in hematoxylin (Mayer’s, Merck, 3 min) and eosin Y (2 min) solution, dehydrated, mounted in Permount, and imaged with a bright field microscope (Olympus IX83 epifluorescent microscope). Histological sections were analyzed by a non-blinded clinical cardiac pathologist with expertise in assessing for morphological irregularities and necrotic tissue who was asked to determine the heterogeneity of the cardiac spheroids as well as examine for apoptotic or necrotic regions. Heterogeneity was defined as structural and size differences between cells in different regions of the spheroid.

### Immunostaining

OCT-embedded sections (10-μm) and chamber slides containing live cells were fixed for 20 min in 4% PFA; blocked and permeabilized for 30 min in 10% donkey serum, 10% Tween20, 3% BSA, and 0.05% Triton-X; incubated with primary antibodies (Supplementary Table 1) for 1 h at room temperature; washed with PBS (3 washes, 5 min per wash), incubated with fluorescent (4′, 6-diamidino-2-phenylindole [DAPI]) secondary antibodies for 1 h at room temperature; mounted in VECTASHIELD hardset Antifade Mounting Medium; and visualized via confocal laser scanning (Olympus FV3000 confocal microscope). Stains were assessed through visual inspection of at least 4 different spheroids from multiple batches and where used, quantification of TUNEL positive cells was determined via manual counting of all nuclei in a single spheroid cross section.

### Transmission Electron Microscopy (TEM)

Spheroid and monolayer CM cells were dissociated, replated on 0.4-μm pore Transwell Polycarbonate Membranes, and cultured for 7 days; then, the membranes were fixed in 2.5% glutaraldehyde solution for 1 h at 4°C and delivered to the UAB High-Resolution Imaging Facility. Sample blocks were sectioned along the width of the transwells with a diamond knife, and samples were mounted and viewed with a Tecnai Spirit T12 Transmission Electron Microscope. Both monolayer and suspension culture groups consisted of 4 transwells each, with at least 3 subsequent samples per transwell sectioned and imaged. Each group (monolayer vs. suspension) consisted of at least 12 images each. Sarcomere lengths were determined using ImageJ with the line measure tool and all sarcomeres in an image were measured.

### Statistical Analysis

Data are presented as mean ± SEM, and significance was evaluated via the Student’s *t-test* or analysis of variance (ANOVA). Analyses were performed with GraphPad Prism8 software (GraphPad Prism, RRID:SCR_002798), and *p* < 0.05 was considered significant.

## Results

### Optimization of hiPSC-CM Differentiation in Spheroid Suspensions

**FIGURE 1:**
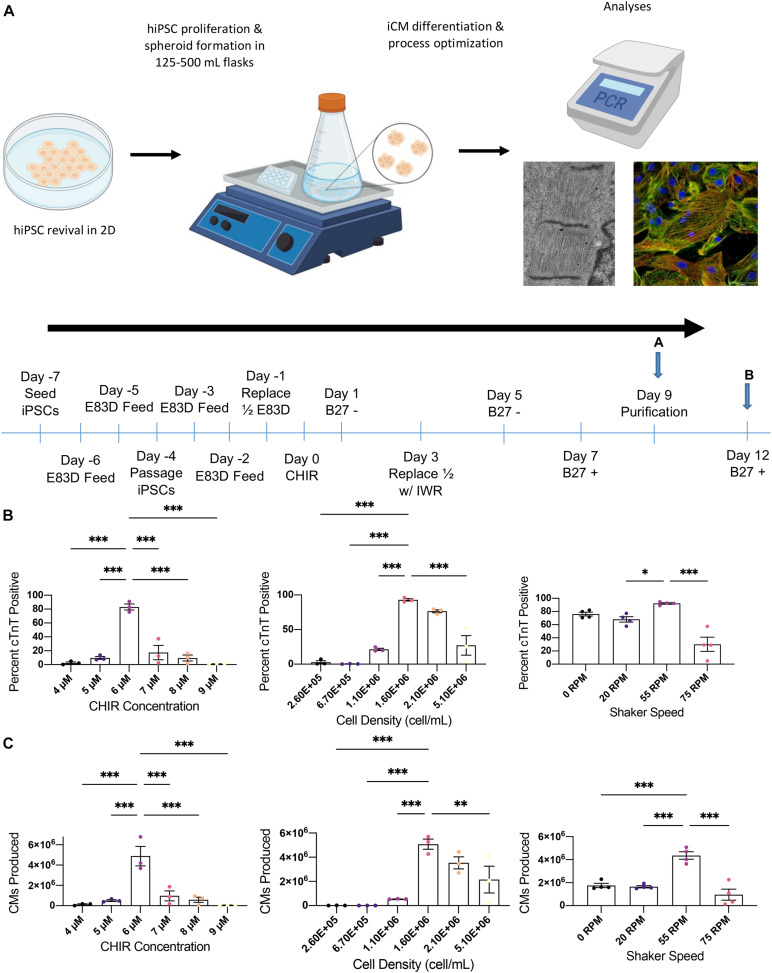
Optimization of suspension differentiation protocol. **(A)** Schematic of the overall process and differentiation timeline for cardiac spheroid production pursued in this paper. Monolayer cultured iPSCs were seeded into 3D culture where they were differentiated followed by multimodal biomolecular and functional analysis. After seeding on day-7 cells are cultured in TeSR E8 3D for 7 days with a single passage on day-4. Cell density is calculated on day 0 and spheroids are then cultured in RPMI1640/B27- supplemented with CHIR on day 1 and IWR on day 3. Metabolic purification is initiated on day 9 using RPMI 1640 without glucose supplemented with B27+ and d-lactate. On day 12 and every 3 days thereafter, media is partially changed with fresh RPMI1640/B27+. Optimization studies analyzed cells at point A and all characterization experiments used cells at point B. **(B)** Flow cytometric analysis of cTnT positive cells for optimization of differentiation conditions including CHIR concentration, cell density on day 0, and shaker speed. **(C)** Total cardiomyocytes produced in each differentiation condition during optimization. ^∗^*p* < 0.05; ^∗∗^*p* < 0.01; ^∗∗∗^*p* < 0.001 (*n* = 4).

hiPSCs were cultured in 6-well plates until 90–100% confluent and then in suspension for 7 days before differentiation was induced by culturing the cells in CHIR99021-containing medium for 24 h beginning on Day 0 and then in IWR1-containing medium for 48 h beginning on day 3 (Figure 1A). The differentiation protocol was conducted in low attachment 6 well plates with rotational shaking, and the protocol was optimized by varying either the cell density (0.26, 0.67, 1.1, 1.6, 2.1, and 5.1 10^6^ cell/mL), CHIR99021 concentration (4, 5, 6, 7, 8, and 9 μM), or shaking speed (0, 20, 55, and 75 rpm) while holding the other 2 parameters constant. Differentiation efficiency was determined on day 9 via flow cytometry measurements of cTnT expression; optimal results for both the purity (Figure 1B) and yield (Figure 1C) of cTNT-positive cells was achieved with an initial cell density of 1.6 10^6^ cells/mL (purity: 92.9 ± 1.8%, yield: 5.08 ± 0.42 10^6^ hiPSC-CMs), 6 μM CHIR99021 (83.1 ± 4.4%, 4.88 ± 0.96 10^6^ hiPSC-CMs), and 55 rpm shaking (92.2 ± 1.2%, 4.36 ± 0.32 10^6^ hiPSC-CMs).

### Characterization of hiPSC-CMs in Suspension-Differentiated Spheroids

**FIGURE 2:**
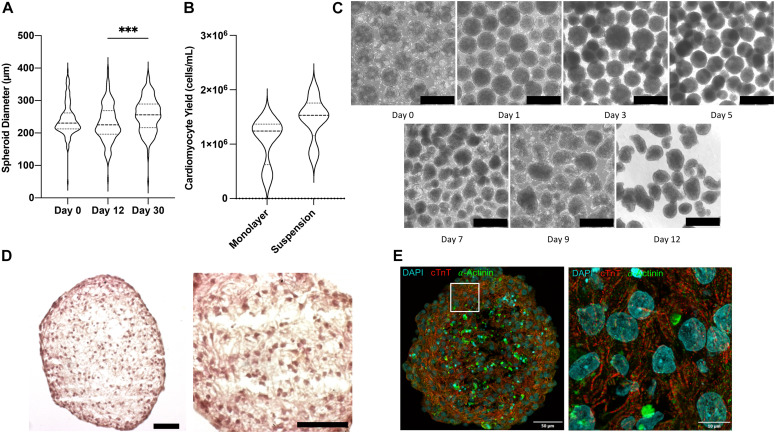
Morphological characterization of CM spheroids. **(A)** Violin plot of spheroid size determined from bright field images on day 0, 12, and 30 of differentiation (*n* > 150). **(B)** Violin plot of cardiomyocyte yield on day 12 in monolayer and suspension culture systems determined as cells per volume of media (*n* = 4). **(C)** Bright field photos of differentiating spheroids over time. Scale bar = 500 μm. **(D)** H&E stained parafin sections of day 12 beating cardiomyocyte spheroids. Scale bar = 50 μm. **(E)** Flourescently stained cryosection showing cardiomyocytes muscle fiber striations. ^∗∗∗^*p* < 0.001.

Shaker flask suspension culture of iPSC aggregates results in a wide range of spheroid sizes and culture heterogeneity (Otsuji et al., 2014). Therefore, prior to beginning differentiation spheroids were passed through a 500 μm filter and collected on a 40 μm filter to remove large aggregates and small debris. When differentiated under optimized conditions, spheroid sizes remained largely stable: mean diameter was 242.4 ± 3.9 μm before differentiation was initiated, 231.4 ± 4.5 μm on day 12 after 30 days of purification via glucose starvation, and 255.2 ± 4.2 μm on day 30 (Figure 2A). Compared with monolayer differentiation (1.08 ± 0.22 10^6^ CM/mL), the suspension protocol produced greater yields of cells (1.47 ± 0.18 10^6^ CM/mL), however, the difference did not reach statistical significance (*p* = 0.203) (Figure 2B). The most conspicuous changes in spheroid morphology were observed on days 1 and 5, after completion of the 24-h CHIR99021 and 48-h IWR1 culture periods, respectively (Figure 2C). Histological (Figure 2D) and immunofluorescent (Figure 2E) analyses conducted on day 12 indicated that the suspension-differentiated spheroids were composed of homogeneous hiPSC-CM populations that were morphologically similar to fetal cardiomyocytes; displayed no evidence of glandular or other cell populations and no large structural irregularities; consistently expressed cardiac troponin T (cTnT) and alpha-actinin; and had moderately aligned, striated fibers with uniformly distributed spherical nuclei.

**FIGURE 3:**
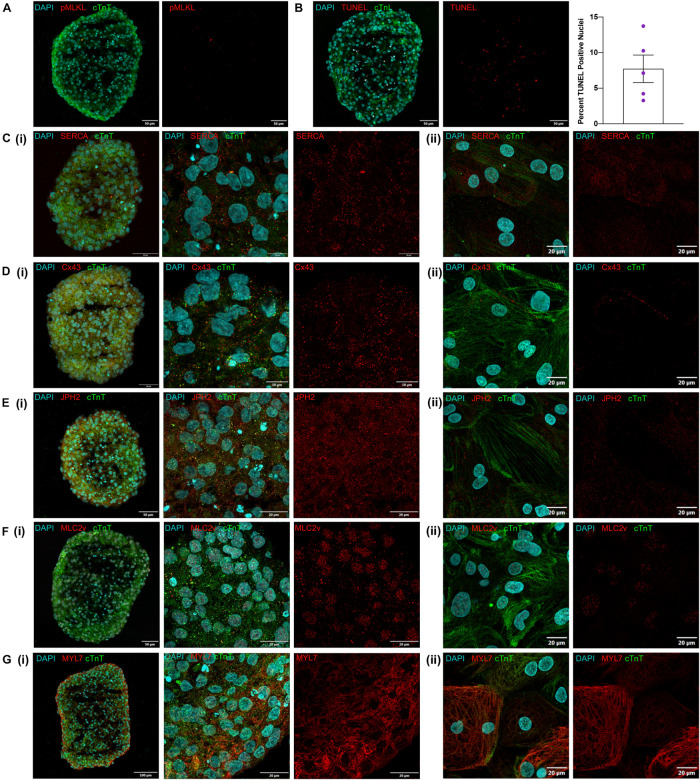
Visual characterization of iCMs produced in suspension. **(A)** Day 12 cryosection stained for pMLKL showing no positive nuclei. **(B)** Day 12 cryosection with TUNEL staining and quantification of percent positive nuclei from examination of 5 spheroids. **(C–G)** Immunofluorescent antibody staining of day 12 suspension cardiomyocytes for cTnT and **(C)** SERCA, **(D)** Cx43, **(E)** JPH2, **(F)** MLC2v, and **(G)** MYL7 as (i) cryosections at low and high magnification and (ii) dissociated spheroids cultured for 3 days as monolayers.

One key concern for spheroid culture is whether cells located in the interior of the spheroid are adequately exposed to nutrients and differentiation factors present in the media; thus, spheroid sections were stained for the expression of phosphorylated mixed lineage kinase domain-like protein (pMLKL) (Linkermann et al., 2014; Negroni et al., 2017) and via terminal deoxynucleotidyl transferase dUTP nick-end labeling (TUNEL) to identify necrotic and apoptotic cells, respectively. None of the sections contained pMLKL-positive nuclei (Figure 3A) and although 7.7 ± 4.3% of cells were TUNEL-positive (Figure 3B) they were evenly distributed throughout the sections rather than localized in the core, which suggests that apoptosis was not caused by lack of access to nutrients in the media. Markers associated with calcium handling and maturation (SERCA, Cx43, JPH2, and MLC2v) were also uniformly expressed throughout spheroid sections and in cardiomyocytes from dissociated spheroids (Figures 3C–G) thus, differentiation appeared to be equally efficient throughout the entire volume of the spheroid, including the spheroid interior. Expression of the atrial isoform marker MYL7, however, was noted to appear higher at the edges of the spheroid than in the internal region. Although initially attributed to greater cell density this observation also presents the possibility that subpopulations of more atrial and ventricular cells may be localized to different regions of the spheroid. Further examination using techniques such as single cell sequencing could help elucidate these differences but were outside the scope of this work.

**FIGURE 4:**
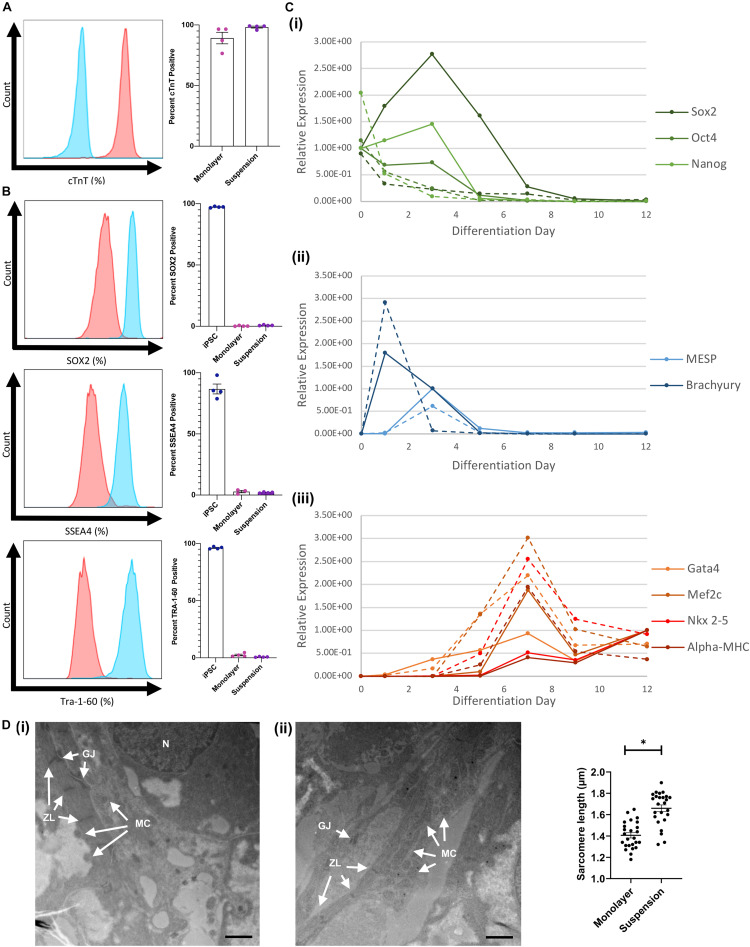
Comparison of monolayer and suspension using the optimized protocol. **(A)** Flow cytometry staining for cTnT in suspension differentiated cardiomyoctes (red) compared with antibody isotype control (blue) with corresponding quantification. **(B)** Flow cytometry staining for iPSC markers (SOX2, SSEA4, and Tra-1–60) in monolayer and suspension (red) differentiated cardiomyoctes compared with iPSC (blue) positive control. **(C)** RT-qPCR analysis of (i) stem cell, (ii) mesoderm, and (iii) cardiac gene expression throughout differentiation for monolayer (solid) and suspension (dotted) techniques. **(D)** TEM images of (i) suspension and (ii) monolayer differentiated CMs with labeled characteristic features, N nucleus, GJ gap junction, ZL z-line, and MC mitochondria (Scale bar = 1 μm). Measurements of mean sarcomere length in each condition (*n* = 25). ^∗^*p* < 0.0001.

Flow cytometry assessments conducted with cells collected on day 12 indicated that cTnT was expressed by a considerably greater proportion of hiPSC-CMs when the cells were differentiated via the suspension protocol (98.2 ± 0.8%) than in monolayers (89.2 ± 4.8%) (Figure 4A) and while expression of the pluripotency markers SOX2, SSEA4, and Tra-1–60 was measurable in suspension-differentiated hiPSC-CMs (SOX2: 0.76 ± 0.18, SSEA4: 2.01 ± 0.22, Tra-1–60 0.75 ± 0.16), the proportion remained at or below that of monolayer-differentiated cells (SOX2: 0.26 ± 0.16, SSEA4: 2.83 ± 0.98, Tra-1–60 2.47 ± 0.74) (Figure 4B). RT-qPCR assessments of mRNA levels indicated that pluripotency gene expression declined immediately and rapidly after suspension differentiation was initiated but peaked on day 3 in monolayer-differentiated cells, while expression of the mesoderm genes Brachyury and MESP peaked on days 1 and 3 (respectively), and cardiac gene expression (Gata4, Mef2c, Nkx2–5, and α-MHC) peaked on day 7, with both protocols (Figure 4C). Peak levels of mesoderm-gene expression in suspension- and monolayer-differentiated cells were similar, but cells differentiated via the suspension protocol tended to express higher levels of cardiac genes, and the monolayer protocol was associated with greater variability between samples for all lineage markers (i.e., pluripotency, mesodermal, and cardiac). Notably, when hiPSC-CMs were imaged via TEM, measurements of mean sarcomere length were significantly greater in suspension-differentiated (1.660 ± 0.155 μm) than in monolayer-differentiated (1.406 ± 0.125 μm) cells (Figure 4D).

### Maturity of hiPSC-CMs in Suspension-Differentiated Spheroids

**FIGURE 5:**
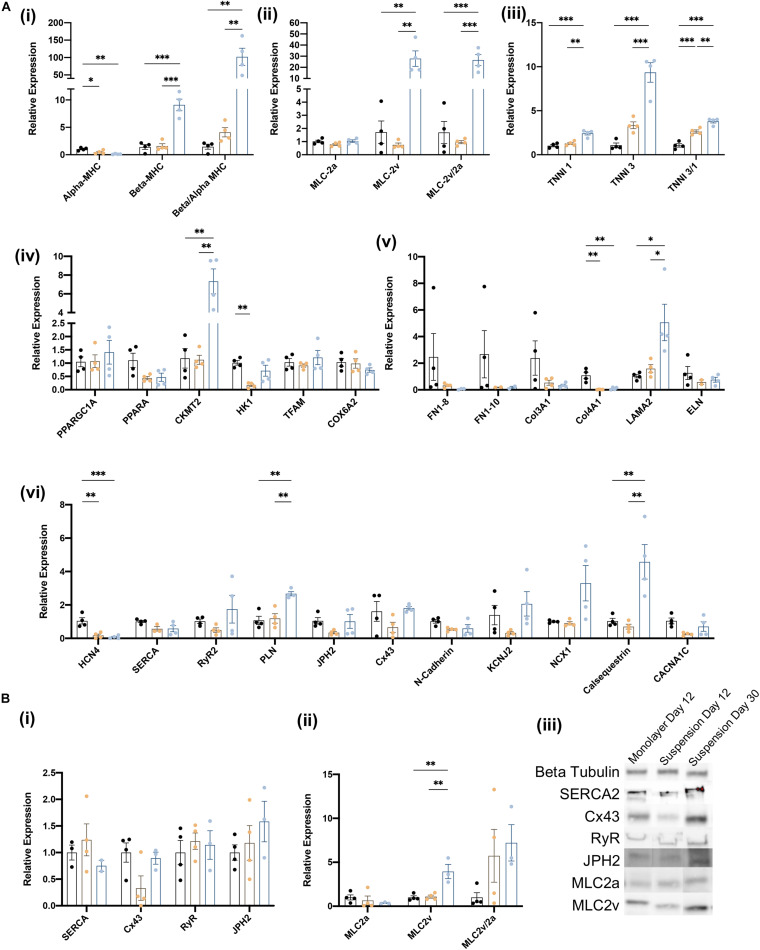
Characterization of iCMs produced in suspension vs. monolayer for biochemical markers of maturation. **(A)** RT-qPCR analysis of relative gene expression normalized to GAPDH and monolayer expression on day 12 and 30 for (i) myosin heavy chain isoforms, (ii) myosin light chain isoforms, (iii) troponin I isoforms, (iv) metabolic activity, (v) structural organization, and (vi) calcium handling genes. Groups are as follows, black: monolayer CMs at day 12, blue: suspension CMs at day 12, and yellow: suspension CMs at day 30. **(B)** Western blot quantification of proteins related to (i) CM maturity and (ii) myosin light chain isoforms. (iii) Blot image for selected samples. ^∗^*p* < 0.05; ^∗∗^*p* < 0.01; ^∗∗∗^*p* < 0.001. (*n* = 4).

The biological processes associated with cardiomyocyte maturation include changes in the composition of the sarcomere, as well as in the expression of genes involved in cellular metabolism, structural organization, and electrophysiology (e.g., calcium handling). Thus, we evaluated the maturity of suspension- and monolayer-differentiated hiPSC-CMs by conducting RT-qPCR assessments of mRNA abundance for individual genes, or the ratio of mRNA abundance for pairs of genes, that typically increase (Beta-MHC, Beta/Alpha-MHC, MLC-2v, MLC-2/2a, TNNI3, TNNI3/1) or decline (Alpha-MHC, MLC-2a, TNNI1) as cardiomyocytes mature (Guo and Pu, 2020). Assessments conducted in cells collected on day 12 indicated that the ratio of βMHC-to-αMHC expression (Mahdavi et al., 1984; Reiser et al., 2001; Yang et al., 2014; LaBarge et al., 2019), as well as both TNNI3 mRNA levels and the TNNI3-to-TNNI1 ratio (Bedada et al., 2014) were greater in suspension-differentiated hiPSC-CMs than in hiPSC-CMs that were differentiated in monolayers (Figure 5A). Furthermore, the same three parameters, as well as MLC2v mRNA levels, the MCL2v-to-MCL2a ratio, and the abundance of CKMT2, LAMA2, PLN, Cx43, NCX1, and Calsequestrin mRNA (Kubalak et al., 1994; Uosaki et al., 2015), increased substantially from days 12 to 30 in suspension-differentiated hiPSC-CMs. Notably, measurements for genes involved in organizational structure (FN1, Col3A1, and ELN) tended to vary more between samples from monolayer-differentiated than suspension-differentiated cells, and the results from Western-blot assessments of protein levels for a subset of key genes were consistent with mRNA measurements (Figure 5B) day 12 measurements in suspension- and monolayer-differentiated cells were similar, while both MLC2v protein levels and the ratio of MCL2v-to-MLC2a protein abundance increased from days 12 to 30. Collectively, these observations confirm that hiPSC-CMs were no less mature when differentiated in suspension than in monolayers.

## Discussion

The GiWi method is among the most efficient strategies for differentiating hiPSCs into cardiomyocytes; however, it may not be sufficiently scalable to produce the billions of hiPSC-CMs needed for treatment of myocardial disease or for high-throughput drug-testing, because it is typically conducted with 2D cell sheets in 6-well culture plates (Sharma et al., 2015). hiPSC-CMs can also be produced in suspension culture (Shafa et al., 2011; Kempf et al., 2014, 2015, 2016; Fonoudi et al., 2016; Halloin et al., 2019; Hamad et al., 2019; Chang et al., 2020; Laco et al., 2020; Miwa et al., 2020), which is more compatible with large-scale production, and the GiWi-based suspension-differentiation protocol introduced here incorporates a number of other key innovations, such as (1) the use of hiPSC culture media that was designed specifically for 3D culture and supplied via a fed batch reactor, (2) a filtration step before differentiation to reduce the heterogeneity of the spheroid population, (3) partial media changes on day 3 and from day 12 onward, which reduced processing time, and (4) direct incorporation of metabolic purification, which increased the purity of the differentiated hiPSC-CM populations to > 98%. Furthermore, whereas newly differentiated hiPSC-CMs are more phenotypically similar to fetal than to adult cardiomyocytes (Xu et al., 2009; Gupta et al., 2010; Yang et al., 2014; van den Berg et al., 2015), our results suggest that at least some markers for cardiomyocyte maturation tended to be more highly expressed in suspension-differentiated than monolayer-differentiated hiPSC-CMs; this observation is consistent with previous reports that 3D culture conditions appear to promote hiPSC-CM maturity (Correia et al., 2018; Beauchamp et al., 2020; Giacomelli et al., 2020). However, for complete assessment of functional maturity, electrophysiology measurements along with longitudinal studies are needed but were outside the scope of the current work.

The efficiency of our differentiation protocol was highly dependent on the initial cell density, CHIR99021 concentration, and shaker speed, but once the optimal conditions were identified, the protocol could be scaled up by ∼10-fold from 3 to 30 mL with no additional optimization. Furthermore, although the efficiencies of the suspension- and monolayer-differentiation protocols were similar, between-batch variation was lower for suspension-differentiated cells, and this consistency across a wide range of culture volumes has important implications for large-scale, commercial biomanufacturing facilities. However, the cumulative evidence from a number of reports suggests that the optimal CHIR99021 concentration can vary depending on which line of hiPSCs is used (Kempf et al., 2015), so our optimization protocol will likely need to be repeated for different hiPSC lines.

In further examining the optimization data, the density dependence most likely results from two competing components, that of limited intercellular communication via soluble factors at low densities and nutrient usage at high densities. This is consistent with our observation that at high seeding densities the final overall cell number was low and significant debris was generated. Additional examination of the concentrations of different signaling factors in the media could help elucidate this further. Cardiac differentiation sensitivity to CHIR concentration has been previously reported and is known to be highly sensitive and variable across cell lines (Lian et al., 2012). Finally, the response to shaker speed variation elucidates a common challenge with pluripotent cell 3D culture that high speeds generate greater sheer stress leading to cell death and low speeds result in aggregation and reduced mixing which negatively effects differentiation potential (Otsuji et al., 2014; Vining and Mooney, 2017). Further scale up and different bioreactor formats will most likely require reassessment of mixing dynamics, but these results provide a framework for determining over or under mixing.

A key limitation and potential for future research is the scalability of the system presented in this work. The simplicity of the shaker flask system with its key advantage in accessibility also provides significant barriers in achieving larger culture volumes. Additional media decreases the surface area to volume ratio requiring advanced gas exchange systems to achieve comparable levels of oxygenation. Further, mass transport dynamics will require alternative vessel formats and mixing such as a stirred tank bioreactor (Kehoe et al., 2010; Olmer et al., 2012). Each of these modifications will require additional engineering and optimization such as the damage that arises with increased shear stress produced by faster impeller speeds. Finally, when considering future clinical applications additional regulatory needs must be met (xeno-free media compatibility and GMP-compliance) as well as manufacturing standardization and automation.

In conclusion, this report introduces an optimized protocol for differentiating suspensions of hiPSC spheroids into cardiomyocytes in a widely available format. Our method produces exceptionally pure (>98%) hiPSC-CM populations with low variation between batches and can function as a groundwork for future bioreactor systems to produce the large number of cells needed for clinical applications.

## Data Availability Statement

The raw data supporting the conclusions of this article will be made available by the authors, without undue reservation.

## Author Contributions

AK-K developed the differentiation protocol, designed the experiments, performed histology and imaging, prepared the sample, and wrote the manuscript. DP assisted in experimental design, sample staining, and imaging. JO performed cell culture and assisted in experimental design. VF and JB reviewed the manuscript. SL assisted with histological processing, imaging, and analysis. XL and JZ provided project leadership, funding acquisition, method development and manuscript revisions. All authors contributed to the article and approved the submitted version.

## Conflict of Interest

The authors declare that the research was conducted in the absence of any commercial or financial relationships that could be construed as a potential conflict of interest.
