# Supplementary material for: Bioreactor Suspension Culture: Differentiation and Production of Cardiomyocyte Spheroids From Human Induced Pluripotent Stem Cells
Source: Front Bioeng Biotechnol. 2021 Jun 11;9:674260. doi: 10.3389/fbioe.2021.674260 (PMC8226172; doi:10.3389/fbioe.2021.674260)
Supplement: Supplementary file 1 [file Table_1.DOCX]

Supplementary Material

1. **Supplementary Video 1**

Beating cardiomyocyte spheroids on day 12 of differentiation produced in a 30 mL culture volume.

1. **Supplementary Table 1-Antibody List**

| **Antibody Name** | **Source** | **Catalog Number** | **RRID** | **Application (FC=Flow Cytometry, I=Immunostaining, WB= Western Blot)** | **Dilution** |
| --- | --- | --- | --- | --- | --- |
| Troponin T, Cardiac Isoform Ab-1, Mouse Monoclonal Antibody | Thermo Fisher | MS295P | AB_61806 | FC | 1:200 |
| Recombinant Anti-Cardiac Troponin T antibody [EPR3695] | Abcam | ab91605 | AB_2050427 | I | 1:100 |
| Monoclonal Anti-α-Actinin (Sarcomeric) | Sigma | A7811 | AB_476766 | I | 1:100 |
| Phospho-MLKL (Ser358) (D6H3V) Rabbit mAb | Cell Signaling | 91689 | AB_2732034 | I | 1:100 |
| Anti-Cardiac Troponin T antibody [1F11] | Abcam | ab10214 | AB_2206574 | I | 1:100 |
| In Situ Cell Death Detection Kit, TMR red | Sigma | 12156792910 |  | I |  |
| Anti-Cardiac Troponin I antibody | Abcam | ab47003 | AB_869982 | I | 1:100 |
| Anti-SERCA2 ATPase antibody | Abcam | ab3625 | AB_303961 | I, WB | 1:100, 1:1000 |
| Anti-Connexin 43 / GJA1 antibody - Intercellular Junction Marker | Abcam | ab11370 | AB_297976 | I, WB | 1:100, 1:1000 |
| JPH2 Polyclonal Antibody | Invitrogen | 40-5300 | AB_2533471 | I, WB | 1:100, 1:1000 |
| Myosin Light Chain 2v (D5I1C) (Cardiac Isoform) Rabbit mAb | Cell Signaling | 12975 | AB_2798075 | I, WB | 1:100, 1:1000 |
| MYL7 Polyclonal Antibody | Invitrogen | PA5-30789 | AB_2548263 | I, WB | 1:100, 1:1000 |
| Anti-Ryanodine Receptor antibody [C3-33] | Abcam | ab2827 | AB_2183052 | WB | 1:1000 |
| β-Tubulin Antibody | Cell Signaling | 2146 | AB_2210545 | WB | 1:1500 |
| Anti-SOX2 antibody | Abcam | ab97959 | AB_2341193 | FC | 1:100 |
| Anti-SSEA4 antibody [MC813-70] | Abcam | ab16287 | AB_778073 | FC | 1:100 |
| Anti-TRA-1-60 (R) antibody [TRA-1-60] | Abcam | ab16288 | AB_778563 | FC | 1:100 |
| Goat anti-Mouse IgG (H+L) Highly Cross-Adsorbed Secondary Antibody, Alexa Fluor Plus 488 | Invitrogen | A32723 | AB_2633275 | Secondary Antibody | 1:100 |
| Goat anti-Rabbit IgG (H+L) Highly Cross-Adsorbed Secondary Antibody, Alexa Fluor Plus 488 | Invitrogen | A32731 | AB_2633280 | Secondary Antibody | 1:100 |
| Goat anti-Rabbit IgG (H+L) Highly Cross-Adsorbed Secondary Antibody, Alexa Fluor Plus 555 | Invitrogen | A32732 | AB_2633281 | Secondary Antibody | 1:100 |

1. **Supplementary Table 2-qPCR Primer List**

| **Target Name** | **Forward Primer** | **Reverse Primer** |
| --- | --- | --- |
| SOX2 | GAGGGCTGGACTGCGAACT | TTTGCACCCCTCCCAATTC |
| OCT4 | CAGTGCCCGAAACCCACAC | GGAGACCCAGCAGCCTCAAA |
| Nanog | TTTGGAAGCTGCTGGGGAAG | GATGGGAGGAGGGGAGAGGA |
| MESP | ACCGTCCCCGCTCCTTC | CAGTCTGCCAAGGAACCACT |
| Brachyury | TGCTCACAGACCACAGGC | AATTGGTCCAGCCTTGGAA |
| Gata4 | TGCCGTTCATCTTGTGGTAG | CCGACACCCCAATCTCG |
| Mef2c | GCCCTGAGTCTGAGGACAAG | AGTGAGCTGACAGGGTTGCT |
| Nkx-2-5 | GCGATTATGCAGCGTGCAATGAGT | AACATAAATACGGGTGGGTGCGTG |
| Alpha-MHC | CTCCGTGAAGGGATAACCAGG | TTCACAGTCACCGTCTTCCC |
| Beta-MHC | ACCAACCTGTCCAAGTTCCG | TCATTCAAGCCCTTCGTGCC |
| MLC-2a | GGAGTTCAAAGAAGCCTTCAGC | AAAGAGCGTGAGGAAGACGG |
| MLC-2v | ACATCATCACCCACGGAGAAGAGA | ATTGGAACATGGCCTCTGGATGGA |
| TNNI 1 | GGTGGATGAGGAGCGATACG | GCTTCAGGTCCTTAATCTCCCTG |
| TNNI 3 | GGAGGACACCGAGAAGGAAAAC | TCAAACTTTTTCTTGCGGCCC |
| PPARGC1A | GCTTTCTGGGTGGACTCAAGT | GAGGGCAATCCGTCTTCATCC |
| PPARA | AGCTGTCACCACAGTAGCTTG | CAGAGTGGGCTTTCCGTGTC |
| CKMT2 | GCTCCGGCTTCAAGACACTC | TGCGCTTGGAGGAAATAGCC |
| HK1 | GACGCACCCACAGTATTCCC | GGAAATGAGCCAGGGTCTCC |
| TFAM | CGCTCCCCCTTCAGTTTTGTG | AATCAGGAAGTTCCCTCCAACG |
| COX6A2 | CATCCGCACCAAGCCCTAC | CCTTTATTGTGTCCGGGGGC |
| FN1-8 | TCGTGCTTTGACCCCTACAC | CGGGAATCTTCTCTGTCAGCC |
| FN1-10 | AAGAAGGGCTCGTGTGACAG | TCTTGTCCTACATTCGGCGG |
| Col3A1 | GGATGGTTGCACGAAACACAC | GGTAGTCTCACAGCCTTGCG |
| Col4A1 | GGCAGATTCGGACCACTAGG | GCGTCTGTGGCAATACTAGC |
| LAMA2 | ACTTGAGTATGAAAGCAAGGCCAG | GGAGAGCTCCACAAAACCAGG |
| ELN | GTGTCTGCAGGTGCGGTG | CTGGGTATACACCTGGCAGC |
| HCN4 | CCCGGAGGCCGAGGT | TCAGGTCCCAGTAAAATCTGAAGTC |
| SERCA | TCACCTGTGAGAATTGACTGG | AGAAAGAGTGTGCAGCGGAT |
| RyR2 | TTGGAAGTGGACTCCAAGAAA | CGAAGACGAGATCCAGTTCC |
| PLN | ACAGCTGCCAAGGCTACCTA | GCTTTTGACGTGCTTGTTGA |
| JPH2 | CCAAGTATGAGGGCACCTGG | GCCTTGGTACGTCCCTCCAT |
| Cx43 | GGTGACTGGAGCGCCTTAG | GCGCACATGAGAGATTGGGA |
| N-Cadherin | AGCCAACCTTAACTGAGGAGT | GGCAAGTTGATTGGAGGGATG |
| KCNJ2 | TGCGCCAGCAACAGGACAT | GTGTCTCTGGGAGCCTTGTG |
| NCX1 | CTGGAATTCGAGCTCTCCAC | ACATCTGGAGCTCGAGGAAA` |
| Calsequestrin | GTTGCCCGGGACAATACTGA | CTGTGACATTCACCACCCCA |
| CACNA1C | TGATTCCAACGCCACCAATTC | GAGGAGTCCATAGGCGATTACT |
| GAPDH | GTGGACCTGACCTGCCGTCT | GGAGGAGTGGGTGTCGCTGT |
